# Phenotypic Characterization and Fine Mapping of a Major-Effect Fruit Shape QTL *FS5.2* in Cucumber, *Cucumis sativus* L., with Near-Isogenic Line-Derived Segregating Populations

**DOI:** 10.3390/ijms232113384

**Published:** 2022-11-02

**Authors:** Yupeng Pan, Birong Chen, Lijun Qiao, Feifan Chen, Jianyu Zhao, Zhihui Cheng, Yiqun Weng

**Affiliations:** 1 Department of Horticulture, University of Wisconsin-Madison, Madison, WI 53706, USA; 2 College of Horticulture, Northwest A&F University, Yangling 712100, China; 3 USDA-ARS Vegetable Crops Research Unit, 1575 Linden Dr., Madison, WI 53706, USA

**Keywords:** cucumber, fruit shape, QTL cloning, near-isogenic lines, marker-assisted selection, auxin signaling

## Abstract

Cucumber (*Cucumis sativus* L.) fruit size/shape (FS) is an important yield and quality trait that is quantitatively inherited. Many quantitative trait loci (QTLs) for fruit size/shape have been identified, but very few have been fine-mapped or cloned. In this study, through marker-assisted foreground and background selections, we developed near-isogenic lines (NILs) for a major-effect fruit size/shape QTL *FS5.2* in cucumber. Morphological and microscopic characterization of NILs suggests that the allele of *fs5.2* from the semi-wild Xishuangbanna (XIS) cucumber (*C*. *s*. var. *xishuangbannesis*) reduces fruit elongation but promotes radial growth resulting in shorter but wider fruit, which seems to be due to reduced cell length, but increased cellular layers. Consistent with this, the NIL carrying the homozygous XIS allele (*fs5.2*) had lower auxin/IAA contents in both the ovary and the developing fruit. Fine genetic mapping with NIL-derived segregating populations placed *FS5.2* into a 95.5 kb region with 15 predicted genes, and a homolog of the Arabidopsis *CRABS CLAW* (*CsCRC*) appeared to be the most possible candidate for *FS5.2*. Transcriptome profiling of NIL fruits at anthesis identified differentially expressed genes enriched in the auxin biosynthesis and signaling pathways, as well as genes involved in cell cycle, division, and cell wall processes. We conclude that the major-effect QTL *FS5.2* controls cucumber fruit size/shape through regulating auxin-mediated cell division and expansion for the lateral and longitudinal fruit growth, respectively. The gibberellic acid (GA) signaling pathway also plays a role in *FS5.2*-mediated fruit elongation.

## 1. Introduction

Cucumber (*Cucumis sativus* L.), is an important vegetable crop cultivated worldwide that is eaten either fresh or processed (pickle). Major market classes of cucumber with significant commercial production around the world include Chinese Long, North American slicer and pickle, European greenhouse (Dutch cucumber) and pickling, and Beit Alpha (Mini or Mediterranean type) cucumbers [1,2]. Cucumber is consumed immature, harvested 10–15 days after anthesis when the fruit is at its premium taste/brining quality. This is coincident with its exponential fruit growth, and its size can be significantly affected by environmental factors. In some market groups, during harvest, any fruit outside the commercially accepted size range has to be discarded, which may result in significant yield loss. This is especially true in the once-over machine harvest system that is popular in the US cucumber production. Thus, fruit size is not only an important fruit quality trait; it also directly affects cucumber yield, as well as economic returns and profitability.

Cucumber fruit displays wide variation in size from less than 5 cm in length in the wild cucumber (*C*. *s*. var. *hardwickii*) to more than 60 cm in some East Asian landraces. The fruit shape of cucumber can vary from slightly flat, ellipsoid, obovoid, and round to long or extremely long [3]. Fruit size and shape (FS) of cucumbers could be assessed by fruit length (FL), diameter (FD), or the length-by-diameter ratio (fruit shape index, FSI) [1], which is controlled by quantitative trait loci (QTLs). Nearly 200 FL, FD, or FSI QTLs have been documented in cucumber (reviewed in [3,4]). According to a synopsis of the literature, about 20 consensus fruit size and shape (FS) QTLs have been proposed to explain observed fruit size and shape variation in natural cucumber populations [3,5]. Nevertheless, our knowledge on molecular regulation of fruit size/shape variation in cucumber is still limited. Despite the large number of FS QTLs identified, very few have been cloned. Three EMS mutants, *short fruit-1* (*sf-1*, *SF1*), *short fruit-2* (*sf-2*, *SF2*), and *short fruit-3* (*sf-3*), were shown to encode a cucurbit-specific RING-type E3 ligase, a histone deacetylase complex 1 (HDC1) protein, and a katanin p60 subunit (CsKTN1), respectively [6,7,8]. In natural cucumber populations, the *FRUITFULL-like* MADS-box gene (*CsFUL1*) plays an important role in fruit elongation in Chinese Long cucumbers [9]. Pan et al. [10] conducted QTL mapping on fruit size variation in segregating populations derived from a cross between WI7238 (long fruit) and WI7239 (round fruit), and they found that the round fruit shape in WI7239 is controlled by two QTLs, *FS1.2* and *FS2.1*, which encode the tomato SUN homolog (CsSUN) and the SlTRM5 homolog (CsTRM), respectively [10,11]. Overall, cloning of FS QTL in cucumber remains a challenge because multiple QTLs underlie fruit size/shape variation in most populations examined.

Marker-assisted near-isogenic line (NIL) development is a powerful tool to Mendelize target QTL for subsequent cloning and functional characterization, which is especially efficient and time-saving for recessively inherited traits. Some successful examples to clone QTL with NIL-derived segregating populations include cloning of the grain size QTL *GS3* [12], *GS5* [13], *qGL3* [14], *GL7* [15], and grain weight QTL *GW2* [16] in rice, fruit shape QTL *sun* [17] and fruit weight QTL *fw3.2* in tomato [18,19], oil content QTL *OilA1* in rapeseed [20], kernel row number QTL *qKRN5b* in maize [21], fiber length QTL *qFL-chr1* [22], and *Fusarium* head blight resistance QTL *Fhb7* in wheat [23]. In cucumber, Xu et al. [24] fine-mapped a powdery mildew (PM) resistance major-effect QTL *Pm1.1* with a single-segment substitution line SSSL0.7, which is also an NIL of the locus.

In an early study, using an F_2:3_ mapping population derived from the cross between semi-wild XIS cucumber (*C*. *s*. var. *xishuangbannesis*) WI7167 and primitive cultivated cucumber WI7200, we identified eight fruit size QTLs (*FS1.1*, *FS1.2*, *FS2.1*, *FS3.2*, *FS4.1*, *FS5.2*, *FS6.3*, and *FS7.1*) [25]. Among them, *fs5.2* exhibited the largest effect on the determination of round fruit shape that is characteristic of WI7167 [25]. However, the exact roles of *FS5.2* in fruit size/shape development and the underlying gene of this locus are unknown. Therefore, the objectives of this study were to conduct fine mapping of *fs5.2* and investigate its effects on fruit growth. We developed NILs for *fs5.2* through marker-assisted foreground (the genomic region containing target gene or QTL from the donor) and background (all the genomic regions carried by the recipient parent) selections. We conducted comparative morphological, microscopic, phytohormonal, and transcriptomic analyses at different fruit development stages of the NILs. Using NIL-derived segregating populations, we fine-mapped the *fs5.2* QTL to a 95.5 kb region. A candidate gene for *fs5.2* was also proposed.

## 2. Results

### 2.1. Development and Phenotypic Characterization of FS5.2 NILs

**Figure 1:**
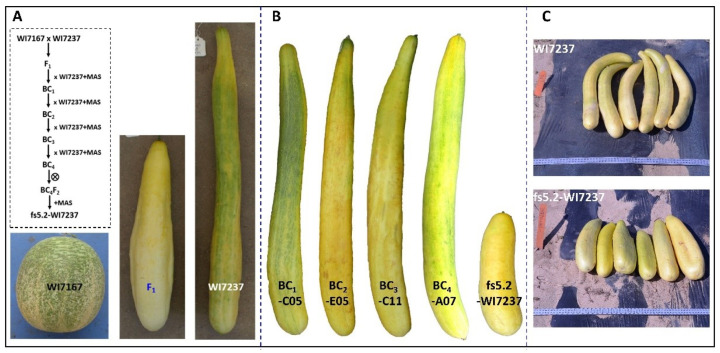
Marker-assisted development of NILs for major-effect QTL *FS5.2* for fruit size/shape. (**A**) Appearances of fruits of *fs5.2* donor WI7167, recipient WI7237, and their F_1_. Flow chart of NIL development is shown in inset. (**B**) Fruit appearances of BC_1–4_ (heterozygotes) and fs5.2-WI7237 (homozygous) NIL. (**C**) Mature fruits of WI7237 and fs5.2-WI7237 under field conditions at HARS, Wisconsin. MAS = marker-assisted selection.

Using segregating populations derived from the round-fruited, semi-wild XIS cucumber W7167, we previously identified four moderate- or major-effect fruit size/shape QTLs *FS1.1*, *FS3.2*, *FS5.2*, and *FS6.3*, among which *FS5.2* had the strongest effect on round fruit shape [3,25]. For fine mapping of *FS5.2*, we first developed NILs to Mendelize this QTL through marker-assisted backcrossing (MABC) by introducing the *fs5.2* allele from WI7167 into WI7237, which bears very long and slim fruit (mature fruit length >50 cm, Figure 1A). The WI7167 × WI7237 F_1_ was backcrossed with WI7237 for four generations (BC_1–4_). Starting from BC_1_, both foreground (for target QTL) and background selections were practiced (Figure 1A). Foreground selection was performed with three markers: the peak marker 8G01 associated with this QTL, and two flanking markers 97C12 and 98C06 which were ~1.7 Mbp apart (Supplementary Table S1). Among 48 BC_1_ plants genotyped with the two flanking markers, 19 were heterozygous at the *fs5.2* locus. To make sure that the selected BC_1_ plant did not carry WI7167 alleles for the other three QTLs (*fs1.1*, *fs3.2*, and *fs6.3*), we employed nine SSR or Indel markers tagging these QTLs to genotype the 19 selected BC_1_ plants (Table S1), and we identified one plant, BC_1_-C05 that was heterozygous at *fs5.2* locus, and homozygous at all other three loci marker for WI7237 alleles, which was backcrossed with WI7237. Among the 192 BC_2_ plants, foreground selection identified 86 target individuals, and background checking was performed with 41 polymorphic markers (Table S1), resulting in plant BC_2_-E05 having the most (21 out of 41; Table S1) marker-based genetic background of WI7237. From foreground selection of 96 BC_3_ plants, 35 target individuals were selected, of which, the single plant, BC_3_-C11, with the most WI7237 backgrounds (at 17 of 20 marker loci; Table S1) was backcrossed with WI7237. Lastly, from 48 BC_4_ plants subjected to foreground and background selections, BC_4_-A07, which was heterozygous at the three marker loci used for foreground selection and homozygous for the three remaining marker loci from WI7237, was self-pollinated to generate the BC_4_F_2_ population. The resulting plants homozygous at the *fs5.2* locus (*fs5.2fs5.2*) were considered near isogenic with WI7237 (*FS5.2FS5.2*) and designated as fs5.2-WI7237 hereinafter. On the basis of positions of markers used in foreground and background selections (Table S1), the size of the introgressed fragment from WI7167 in fs5.2-WI7237 was approximately 6.2 Mb.

**Figure 2:**
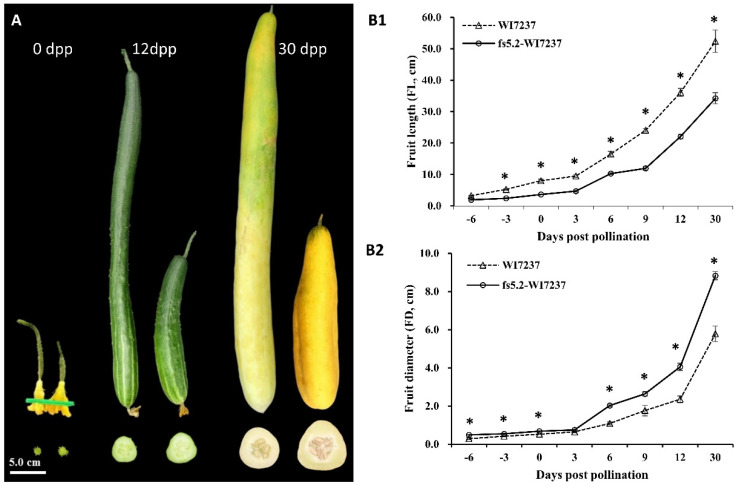
Fruit growth at different development stages of *fs5.2* NILs. (**A**) Appearances of ovaries, immature fruits, and mature fruits of WI7237 (**left**) and fs5.2-WI7237 (**right**) at anthesis day (0 day post pollination, dpp), 12, and 30 dpp. (**B1**,**B2**) Fruit length and diameter at different days of pollination in the NILs. Error bar for each data point represents the mean ± SD (standard derivation) from three replications. At each timepoint, WI7237 fruit is significantly longer but slimmer than that of fs5.2-WI7237 (* *p* < 0.05 based on *t* test).

Fruit images of selected plants at each BC generation are shown in Figure 1B. Fruits from BC_1–4_ plants (range 42–55 cm) which were heterozygous at *fs5.2* locus had similar fruit length as WI7237 (mean ~57 cm), but fs5.2-WI7237 had significantly shorter but wider mature fruit than WI7237 under both greenhouse (Figure 1B) and field conditions (Figure 1C). This is consistent with our early QTL mapping study [25] that the *fs5.2* short allele from WI7167 is recessive to the long allele in WI7237 for this QTL, which inhibits fruit elongation but promotes radial growth (increase fruit diameter). Indeed, we measured the length and diameter of the ovary, immature fruit, and mature fruit of WI7237 and fs5.2-WI7237 at 0, 12, and 30 dpp, respectively (Figure 2A,B) under greenhouse conditions. At each stage, the ovary and fruit of fs5.2-WI7237 were significantly shorter but wider than WI7237. We further measured ovary/immature fruit length and diameter of the two NILs every 3 days from 6 days before anthesis to 12 dpp. At each timepoint, WI7237 always had longer ovary/fruit length but narrower diameter than fs5.2-WI7237 except at 3 dpp. The effect of *fs5.2* on fruit growth could be seen as early as 6 days prior to anthesis (−6 dpp), and the difference in fruit length and diameter became more pronounced 3 days after pollination (Figure 2B). We also introgressed *fs5.2* allele from WI7167 into other cucumber genotypes such as the North China type WI7238 [10], or WI7200, a landrace from Thailand [26], and observed similar effects of *fs5.2* on fruit growth, suggesting that *fs5.2* is effective in different genetic backgrounds.

### 2.2. Microscopic Investigation of Fruit Growth in fs5.2-WI7237 and WI7237 NILs

**Figure 3:**
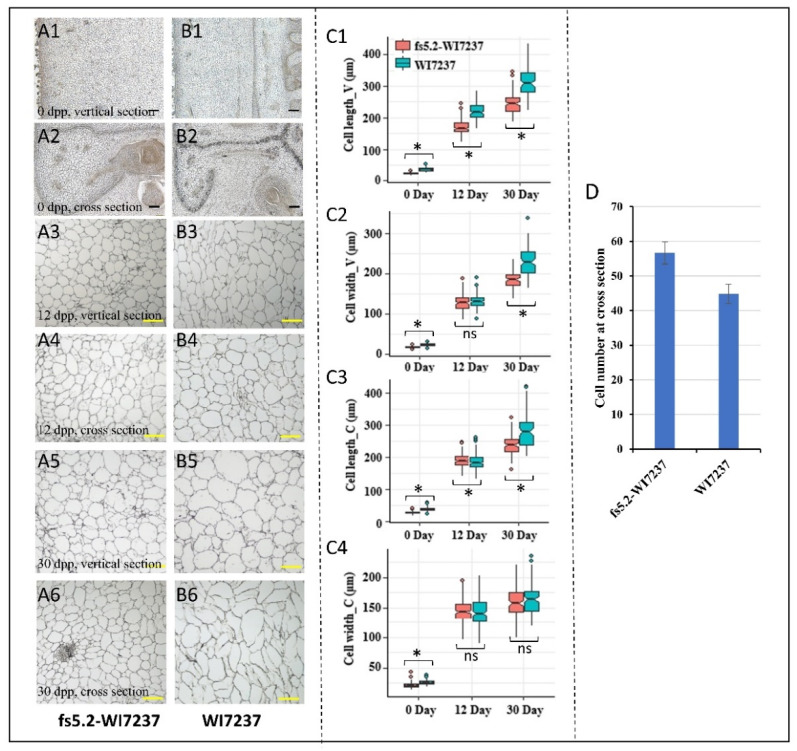
Microscopic images of longitudinal sections (1, 3, and 5) or cross-sections (2, 4, and 6) of ovary/fruit of fs5.2-WI7237 (**A1**–**A6**) and WI7237 (**B1**–**B6**) at different stages. Black bar = 100 μm; yellow bar = 200 μm. (**C**) Cell length and width of vertical sections (V) and cross-sections (C) of WI7237 and fs5.2-WI7237 ovary/fruit at different development stages. * means significantly different at *p* < 0.01; ns = no significant difference. (**D**) Cell numbers (layers) at cross-section of WI7237 and fs5.2-WI7237 ovary at 0 dpp.

We conducted paraffin sectioning of fruit samples of both NILs at three development stages (0, 12, and 30 dpp), and we measured cell length and width at both vertical (longitudinal) and cross (horizontal) sections of the fruit (Figure 3A–C). In the vertical sections, cells of WI7237 were longer than those of fs5.2-WI7237 at all three stages (Figure 3C1); the WI7237 cells were also wider than those of fs5.2-WI7237 at both 0 and 30 dpp, and no difference was observed at 12 dpp (Figure 3C2). At the cross-sections, WI7237 consistently exhibited longer cells than fs5.2-WI7237 throughout the whole development stages (Figure 3C3); cells of WI7237 ovary were wider than those of fs5.2-WI7237, but no difference was found in cell width at 12 and 30 dpp between the two lines (Figure 3C4). We counted the number of cross-section cell layers from the endocarp to exocarp (fruit skin) of ovaries at anthesis (0 dpp) and found that there were more cell layers in fs5.2-WI7237 than in WI7237 (Figure 3D). These observations suggest that *FS5.2* regulates fruit growth through promoting cell elongation longitudinally, as well as cell division horizontally.

### 2.3. Phytohormone Dynamics in Ovary/Fruit Development in NILs

**Figure 4:**
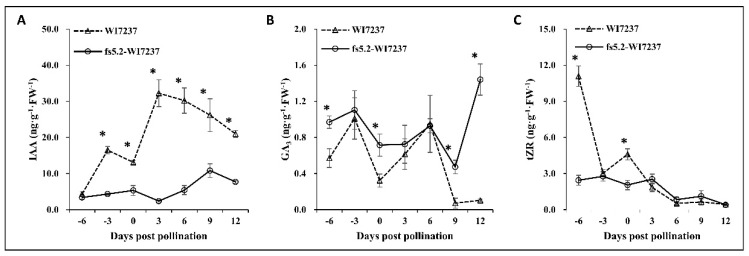
Dynamic change of endogenous phytohormones IAA/auxin (**A**), GA_3_ (active GA, (**B**)), and tZR (active cytokinin, (**C**)) at different ovary/fruit development stages in NIL fruits. Asterisks (*) indicate significant differences between WI7237 and fs5.2-WI7237 at *p* < 0.05 based on *t*-tests.

We investigated the dynamics of auxin (IAA), gibberellin (GA_3_), and cytokinin (tZR) in the ovaries and developing fruits of the two NILs. Ovary/fruit samples were collected at −6, −3, 0, 3, 6, 9, and 12 days post pollination (dpp), which were corresponding to the timepoints for fruit size measurements described above (Figure 2B). Endogenous hormone levels at the seven timepoints in the ovary/immature fruit of the two NILs are illustrated in Figure 4. The contents of the three hormones showed different dynamics during early fruit development. At −6 dpp, IAA content in both NILs was the same. From −6 to 12 dpp, in WI7237, IAA content showed fluctuations with the first and second peaks at −3 and 3 dpp, respectively. This seemed to coincide with cell proliferation/division of ovary/fruit growth. IAA content in the short-fruited NIL (fs5.2-WI7237) also showed fluctuations, but the magnitude of changes over time was much smaller than in WI7237; the peak date was also lagged behind (0 and 9 dpp) as compared with WI7237 (Figure 4A). In addition, except at −6 dpp, IAA contents in WI7237 were always significantly higher than those in fs5.2-WI7237 in any day examined (Figure 4A). These data suggest that IAA plays a critical role in *fs5.2*-medited fruit elongation in WI7237, especially during the early fruit development stage when cell division is most active.

The content of active GA (GA_3_) in both WI7237 and fs5.2-WI7237 also exhibited fluctuations from −6 to 12 dpp but with different patterns from IAA. Similar to IAA, GA_3_ contents showed peaks at −3 and 6 dpp in both NILs; however, in WI2737, GA_3_ started to decline to trace amount at 9 and 12 dpp, while, in *fs5.2*, there was a dramatic increase from 9 to 12 dpp (~3 folds). Unlike IAA, GA_3_ content in fs5.2-WI7237 with shorter but wider fruit was significantly higher than in WI7237 at −6, 0, 9, and 12 dpp (Figure 4B). These observations suggest that GA_3_ may play a more important role in cell expansion for horizontal fruit growth.

The trans-zeatin riboside (tZR) is the most active cytokinin (CK). At −6 dpp, tZR in WI7237 was significantly higher than in fs5.2-WI7237 at −6 dpp. In WI7237, tZR showed a sharp decrease from −6 to −3 dpp, and then a slight increase from −3 to 0 dpp (Figure 4C). In both NILs, tZR kept declining from 0 to 12 dpp. Overall, these data suggest that IAA/auxin plays an important and positive role in fruit set/growth in the NILs throughout the development stages, whereas tZR and GA_3_ play certain roles at ovary development (−6 and 0 dpp) or fruit elongation in late stages (9 and 12 dpp). The dynamic changes of fruit size/shape and endogenous hormones in the two NILs also suggest that *FS5.2* regulates cucumber fruit size/shape from the early stage of ovary development.

### 2.4. Fine Mapping of FS5.2 in NIL-Derived Segregating Populations

**Figure 5:**
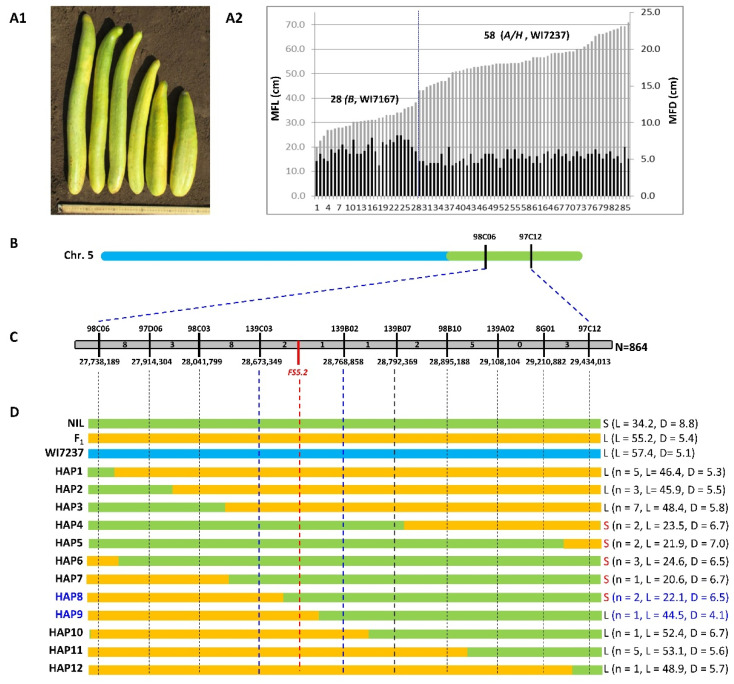
Fine mapping of *FS5.2* major-effect QTL. (**A1**) Representative fruits from NIL-derived F_2_ plants showing segregation of fruit size/shape in this population. (**A2**) Distribution of mature fruit length (MFL) and diameter (MFD) among 82 F_2_ plants based on field data. Genotype of each plant at the *fs5.2* locus was also shown. (**B**–**D**) From 864 F_2_ plants, 33 recombinants were identified with flanking markers 98C06 and 97C12, which were phenotyped and genotyped with eight additional markers (**C**). Twelve haplotypes (HAP) could be recognized, which allowed mapping of *FS5.2* into a 95.5 kb region with 15 predicted genes (**D**). In (**D**), S = short fruit, L = long fruit, and n = # recombinants with the specific haplotype. The light-green, light-blue, and orange bars indicate the genotypes of NIL, WI7237, and F_1_, respectively. L and D are mean fruit length and diameter, respectively, of the haplotype (in cm).

The NILs we developed above effectively Mendelized the *fs5.2* QTL. For fine mapping, a secondary BC_4_F_2_ population segregating for *fs5.2* was developed from MABC progeny (Figure 1). We first tested the inheritance of the *fs5.2* QTL using a small F_2_ population with 94 plants grown in the open field of HARS (Hancock, WI, USA). These plants were genotyped with three markers (97C12, 98C06, and 8G01) used in foreground selection. Of them, 20, 46, and 28 plants carried homozygous A (WI7237), heterozygous (H), and homozygous B (WI7167) alleles at all three marker loci, respectively, fitting a 1:2:1 expected ratio (*p* = 0.4925 in *χ^2^* test). Two plants showed recombination in this region. Typical fruits are shown in Figure 5A1. The length (MFL) and diameter (MFD) of mature fruits of 86 plants were measured and graphed in Figure 5A2. Using mature fruit length of 40 cm as the cutoff, among the 86 plants, 28 had short and fat fruit (mean MFL = 30.5 cm, MFD = 6.5 cm, LD = 4.8) and 58 had long and slim fruit (mean MFL = 56.0 cm, MFD = 5.2 cm, and L/D = 10.8), which was consistent with the expected 3 (long):1 (short) segregation (*p* = 0.1055 in *χ^2^* test) for both MFL and MFD. All plants with short fruit carried homozygous WI7167 (*B*) alleles, and all long-fruited plants were either *A* (WI7237) or *H* at the three marker loci. Most of the 28 short-fruited F_2_ plants also had large MFD values (Figure 5A2). These observations suggested that the fruit size variation in this population is indeed controlled by a single QTL, *fs5.2*, in a ~6.2 Mbp region on Chr 5, and the WI7167 allele (*B*) increases fruit diameter but reduces fruit length.

To narrow down the *fs5.2* region, we screened 864 F_2_ plants of WI7237 × fs5.2-WI7237 (including 96 from 2017 field trail described above) to identify recombinants between the two flanking markers 98C06 and 97C12 that were ~6.2 Mbp apart (Figure 5B; Table S1). Thirty-three recombinant plants were identified. Seven new indel markers were developed in this interval, which, together with the peak marker 8G01, were used to genotype the 33 plants. The genotypic and phenotypic (mature fruit length, diameter, and L/D) data of these plants are provided in Supplementary Table S2. Twelve haplotypes (HAP1-12) could be defined by the eight markers. Phenotypic data (mean fruit length/L and diameter/D) for each haplotype are presented in Figure 5C. Of the 12 haplotypes, five (#4–8) had short (S) fruit (mean MFL = 22.9 cm, MFD = 6.6 cm, L/D = 3.5), and seven (the rest) had long fruit (mean MFL = 48.7cm, MFD = 5.6, and L/D = 8.8). These data were consistent with those observed in the F_2_ population (Figure 5A). On the basis of the fruit size data of the 12 haplotypes, *FS5.2* was located into a 95.5 kb interval (Gy14v2.0) flanked by two markers 139C03 and 139B02.

In Gy14v2.0, 15 genes were annotated in this 95.5 kb region (listed in Table S3). We generated contig assemblies from PacBio HIFI sequencing for several cucumber lines including the two parental lines (unpublished data), WI7200 and WI7167, that we used for initial QTL mapping of *fs5.2*. We retrieved and aligned the complete 95.5 kb genomic DNA sequences of the two lines and identified 103 variants (SNPs and indels). No polymorphisms were found inside 13 of the 15 genes (Table S3) except *CsGy5G023910* and *CsGy5G023950*. Comparison of gDNA sequences including its promoter region revealed that *CsGy5G023910* harbored a SNP (*A* in WI7167 and *G* in WI7200) inside its exonic region. For *CsGy5G023950*, there were eight SNPs inside its coding region including one in its second exon; there were 16 SNPs and two indels in its promoter region between the two parental lines. We further compared the polymorphisms inside the two genes in other 22 cucumber lines with whole genome or HIF contig assemblies (available at https://www.cucurbitgenomics.org/, accessed on 12 February 2021), and we found that the *A* allele inside *CsGy5G023910* was carried only by the round-fruited WI7167, whereas all other lines carried the *G* allele. Meanwhile, no consistent association of SNP alleles with fruit size/shape was found for sequence variants in *CsGy5G023950* among the 24 cucumber lines compared. These data suggest that *CsGy5G023910*, which encodes the Arabidopsis homolog of CRABS CLAW, is the most possible candidate for the fruit shape QTL *fs5.2* in cucumber (see Section 3).

### 2.5. FS5.2-Dependent Gene Network Regulating Fruit Size/Shape with RNA-Seq

To understand the *FS5.2*-regulated gene network for cucumber fruit size/shape formation, transcriptome profiling was performed using ovary samples of the two NILs collected on the day of anthesis with two biological replications. RNA-seq generated 43.0 to 47.2 million raw reads for each sample per replication, in which 42.7–46.8 million clean reads were obtained after removing low-quality and adapter sequences; 38.0–42.8 million reads were uniquely mapped to the cucumber genome Gy14v2.1 (Supplementary Table S4). We evaluated the quality of the transcriptome data by calculating Pearson correlation coefficients for the two samples between the two reps, which were 0.9935 and 0.9691 between WI7237_Rep1 and WI7237_Rep2 and between fs5.2-WI7237_Rep1 and fs5.2-WI7237_Rep2, respectively. This suggested high quality and reproducibility of the two reps of the RNA-Seq data. Using the false discovery rate of *p* < 0.05 as the cutoff, 437 DEGs were identified in the comparison of fs5.2-WI7237 (short fruit) vs. WI7237 (long fruit) NILs (see Supplementary Table S5 for the complete list). Among the 437 DEGs, 251 and 186 were up- and downregulated in fs5.2-WI7237 as compared with WI7237, respectively (Table S5).

**Figure 6:**
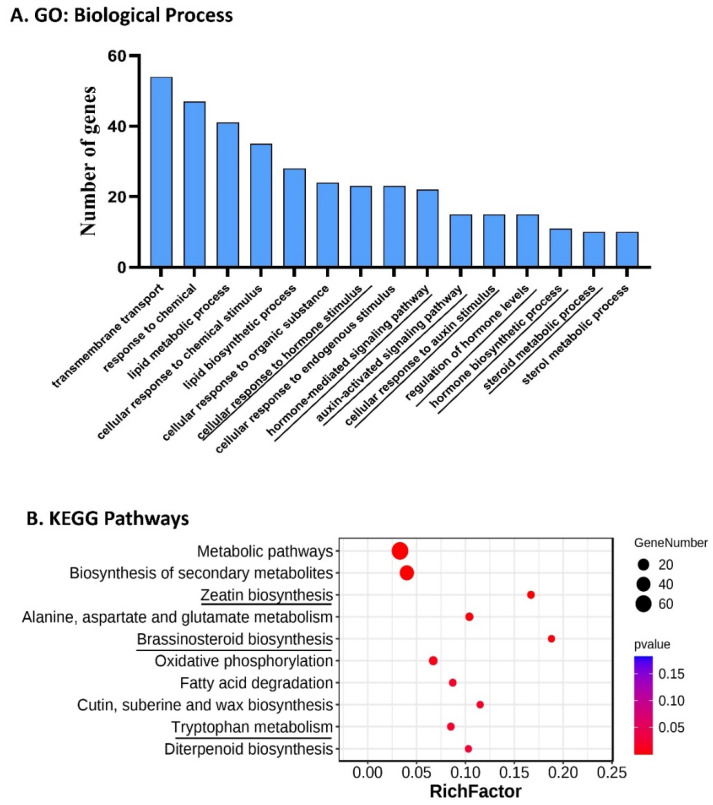
Gene ontology (GO) and KEGG pathway enrichment analyses of transcriptomes of the ovaries at anthesis between WI7237 and fs5.2-WI7237. (**A**,**B**) are significantly enriched biological process and KEGG pathway terms (*p* < 0.05) of the 437 DEGs, respectively.

More than 60 genes for transcription factors were among the DEGs suggesting transcriptional regulation of fruit size and shape in *fs5.2*-dependent ovary/early fruit development. GO and KEGG pathway enrichment analyses indicated that genes involved in hormone biosynthesis/signaling were significantly enriched (Figure 6). Thus, we manually examined all DEGs and highlighted those (total 112) involved in hormone biosynthesis/signaling (38), transcription factors (TFs, 62), or cell division/expansion (23) (Table S5, 11 TFs in the first group). Forty-five and 67 of 112 DEGs were up- and downregulated, respectively, in the short-fruited fs5.2-WI7237 in comparison with the long-fruited WI7237. The 38 hormone-related DEGs included those for biosynthesis/signaling components of all major phytohormones or their crosstalk such as auxin/IAA (nine), gibberellic acid (GA, six), cytokinin (CK, three), brassinosteroid (BR, five), abscisic acid (ABA, six), ethylene (ET, seven), and jasmonate (JA) or salicylic acid (SA) (two). These data suggest that all these hormones play important roles in the *FS5.2*-dependent regulation of fruit size in cucumber. Furthermore, the nine auxin/IAA-related DEGs involved in auxin biosynthesis (*YUCs*), homeostasis (*GH3.6*), signal transduction (*AUXs*), and polar transport (*PIN3* and *PILS7*) supporting the critical roles of auxins in *FS5.2* regulated fruit size/shape, which was consistent with the result in hormone bioassays (Figure 4).

Among the DEGs in Table S5, several have been shown to play important roles in organ size/shape control in Arabidopsis or tomato, including ovate family proteins (OFPs), CYP78A5, cell size regulator (CSR, FAF-like), TCPs, WOX1, YAB5, and several MADS-box transcription factors (Table S5; see Section 3). Regardless of the mechanisms employed, the regulation of final fruit size/shape is achieved through control of the rate, duration, and plane of cell division, as well as the direction and degree of cell expansion. Cell expansion requires concomitant new cell-wall synthesis and loosening of the existing wall. Indeed, the expressions of many genes involved in these processes (cell cycle, division, and cell wall) were also significantly altered between WI7237 and fs5.2-WI7237 (Table S5). Overall, the transcriptomic data revealed a complex picture of the *FS5.2*-mediated gene network in the regulation of fruit size/shape in cucumber.

## 3. Discussion

### 3.1. Mendelization of fs5.2 through NIL Development

In this study, with MABC, near-isogenic lines (NILs) were developed which effectively Mendelized the *fs5.2* major-effect QTL (Figure 1 and Figure 2). The NIL-derived segregating populations allowed fine mapping of *fs5.2* into a 95.5 kb region (Figure 2) and revealed an *fs5.2*-regulated gene network for fruit shape formation (Figure 6; Table S5). NILs are an important tool in plant breeding, fine mapping or cloning of QTLs, and gene function studies [26]. In cucumber, hundreds of QTL for various horticulturally important traits have been identified (e.g., Pan et al. [3]; Wang et al. [4]; Li et al. [27]; Gebretsadik et al. [28]), but very few have been fine-mapped or cloned. Traditional NIL development is very time-consuming. Although marker-assisted backcross (MABC) has greatly accelerated the process of NIL development, how to bring the target allele/gene into a new genetic background quickly and efficiently remains a challenge. Below, we discuss the lessons and experiences we learned from our present and early studies to expedite marker-assisted backcrossing for NIL development in cucumber.

*FS5.2* is a major-effect QTL for fruit size/shape that was identified in an F_2:3_ population derived from the cross between the semi-wild XIS cucumber (WI7167) and a landrace of the cultivated cucumber line WI7200 [25]. Since WI7167 and WI7200 had similar fruit length, and there eight 8 QTLs affecting fruit size/shape in the population, for development of *fs5.2* NILs through MABC, we used WI7237 as the recurrent parent and recipient, which had the longest fruit in our collection (>50 cm, Figure 1; Pan et al. [3]). This would allow eliminating background noises from other QTLs and observe the effect of the WI7167 allele on fruit size/shape. To minimize the genetic background from the donor, ideally, a smaller introgressed fragment carrying the target allele is better. However, since we need to use the resulting NIL pair for fine mapping of *fs5.2*, if the introgressed fragment is too short, it would be very difficult to identify recombinants in the target region for the next-step map-based cloning of *fs5.2*. Thus, we employed three markers for foreground selection at each BC generation; one was the peak marker, and two flanked the 2.0–LOD interval in the initial QTL analysis, all of which were located in a ~2.0 Mbp region (Figure 5, Table S1). In the final generation (BC_4_), the actual introgressions size from WI7167 in fs5.2-WI7237 was ~6.7 Mbp; in this region, adequate numbers of recombinants could be identified during fine genetic mapping (Figure 5; Table S2), which was largely consistent with the recombination rate estimated in this region from the initial QTL mapping study [25]. Thus, for marker-assisted NIL development, the choice of recurrent parent and markers for foreground selection is important when considering the potential uses of the NILs developed.

When multiple QTLs are contributing to phenotypic variation of a trait, it is important to exclude the effect of non-targeting QTLs in NIL development. Here, at BC_1_, nine polymorphic markers (Table S1) were chosen for background selection, which tagged three consensus FS QTLs (*FS1.1*, *FS3.2*, and *FS6.3*) with major or moderate effects on fruit size variation in the WI7167 × WI7200 mapping populations [25]. The first-round background selection effectively eliminated the effects of the WI7167 alleles at non-target loci. This could be evidenced from the very similar fruit length between BC_1_C05 and WI7237, which remained largely unchanged at BC_2_F_1_ through BC_4_F_1_ (Figure 1B). This also suggests that the *fs5.2* allele in WI7167 is recessively inherited. To expedite NIL development, 41 markers were employed for background selection at BC_2–4_. These markers were distributed across the whole cucumber genome except for the target region. At each generation, plants carrying the most homozygous WI7237 alleles of these marker loci were selected (21 at BC_2_, 17 at BC_3_, and three at BC_4_; Table S1) to maximize recovery of the recurrent parent chromatins. Genotyping-by-sequencing (GBS) of the NILs (BC_4_F_2_) indicated that all donor backgrounds in *fs5.2*-NIL were practically replaced by those from the WI7237 recurrent parent except for a ~6.7 Mbp region carrying the *fs5.2* allele of WI7167, suggesting that our MABC strategy was very effective in NIL development for the *fs5.2* QTL. However, retrospectively, we probably could shorten the NIL development by at least one BC generation by screening a larger population at BC_1_ for background selection. The number of markers used in both BC_1_ and BC_2_ (total 50) seem adequate. The BC_2_ population could be increased to 384 instead of 192 plants, which would give a better chance of identifying plants with maximum WI7237 genetic backgrounds. Of course, that requires significantly more effort in genotyping. On the other hand, if more markers are used in background selection at BC_1_, very few markers will be needed at BC_2_ for selection against the reminiscent backgrounds of the donor line, and NILs could be completed as early as BC_2_ F_1_ or BC_3_F_1_.

In cucumber, to shorten the time of NIL development for target QTL, some other practical factors should also be considered. For example, the recipient/recurrent parent should preferably be monoecious to avoid chemical treatment for sex conversion. The line should have relatively short, photoperiod-independent flowering time, and the seeds should have no seed dormancy. Often, multiple QTLs can be tracked in the same population with the MABC scheme, which can be more efficient for QTL pyramiding.

### 3.2. FS5.2 Controls Both Longitudinal and Radial Fruit Growth and Fruit Shape in Cucumber

In our initial QTL mapping study [25], the *fs5.2* QTL showed the strongest association with round fruit shape in the WI7167 × WI7200 F_2_ population. WI7167 bears nearly round fruit (FSI or LD close to 1.0). Interestingly, despite eight QTLs being detected for fruit size/shape variation, among the F_2_, the segregation of plants with non-round to round fruits fit to 3:1, suggesting a single recessive gene may be underlying the ‘round fruit’ locus in this population. Consistent with this, among five LD QTLs detected, *FS5.2* (*ld5.1*) had the highest LOD support (LOD > 40.0) with the strongest (*R*^2^ = 36.7–51.0%), but negative additive effect, which was colocalized with the ‘*round fruit*’ locus from linkage analysis [25]. In addition, the WI7167 alleles at the major-effect QTL *mfl5.1* (for mature fruit length) and *mfd5.1* (for mature fruit diameter) loci had negative (reduction of fruit length) and positive (increase of diameter) additive effects, respectively. *FS5.2*, *mfl5.1*, and *mfd5.1* were all mapped at the same location on cucumber Chromosome 5, strongly suggesting that the WI7167 allele of *FS5.2* inhibits fruit elongation but promotes radial growth and is a true fruit shape QTL. In this study, the *fs5.2* allele from WI7167 was introduced into a different genetic background (WI7237). The resulting NIL fs5.2-WI7237 carrying homozygous *fs5.2* allele showed significantly shorter but wider ovary/fruit from pre-anthesis fruit set, elongation, and maturity, providing convincing evidence to support the roles of *FS5.2* in fruit growth as a fruit shape QTL in different genetic backgrounds.

Over 200 QTLs have been identified to explain fruit size, shape, or fruit weight variation in natural populations of cucumber, from which more than 20 consensus fruit size/shape QTLs have been inferred (reviewed in Weng et al. [2]; Pan et al. [3]). Many of the consensus fruit length or diameter QTLs are colocalized with fruit shape (FSI) QTLs, implying that these QTLs, like *fs5.2*, control fruit shape by regulating both fruit length and diameter. Interestingly, several short-fruit genes (*sf-1*, *sf-2*, and *sf-3*/*CsKTN1*) have been cloned in cucumber; EMS-induced mutations in these genes are responsible for the short fruit phenotypes, which only affect fruit length but have no effect on fruit diameter [6,7,8]. However, a different allelic mutation inside *CsKTN1* encoding a katanin p60 subunit may have a pleiotropic effect on the size of other organs of the cucumber plants [29]. In tomato and several other crops, a number of genes controlling fruit size/shape have been cloned, including those from the OVATE family proteins (OFP) and the SUN gene; often, these genes also control the shape of other organs (e.g., cotyledons, seeds, and flowers) (reviewed in Pan et al. [3]; see below). In this study, other than the fruit size/shape, we did not observe any visible significant differences in the size of other organs between WI7237 and fs5.2-WI7237. Thus, our work seems to be the first major-effect QTL in cucumber with empirical evidence that it controls fruit shape by inhibiting fruit elongation but increasing radial growth which does not seem to affect the shape and size of other organs.

Fruit size/shape variation can be ultimately attributed to the underlying genes that regulate the timing, magnitude, duration, and plane of cell division, as well as isotropic and anisotropic cell enlargement during fruit growth [3,30,31,32]. The comparative microscopic examination of ovary/fruit structure may give us some clues on the differential fruit growth between the NILs (Figure 3). Throughout the development stages of the ovary/fruit, in both longitudinal and cross-section views, cells of WI7237 were longer than those of fs5.2-WI7237; WI7237 had wider than or the same width cells as the *fs5.2*-WI7237 NIL depending on the views or development stages (Figure 3C1–C4), indicating that *FS5.2* promotes fruit elongation through an increase in cell length. However, at the cross-section, there were more cell layers in fs5.2-WI7237 than in WI7237 (Figure 3D), suggesting that an increase in horizontal cell division may contribute to the increase of fruit diameter in fs5.2-WI7237 NIL. Additional work is needed for more insights into the cellular basis of fruit size variation between the NILs.

### 3.3. FS5.2 Putatively Encodes CRAB’s CLAW (CsCRC), a Member of the YABBY Protein Family

Using NIL-derived segregating populations, we fine-mapped *FS5.2* into a 95.5 kb region, in which 15 genes were annotated in Gy14v2.0 (Figure 5; Table S3). Alignment of genomic sequences of the 95.5 kb region between WI7200 and WI7167 revealed polymorphisms in only two of the 15 genes, *CsGy5G023910* and *CsGy5G023950*, which might be the potential candidates of *FS5.2*. However, despite the high level of polymorphisms inside the coding (eight SNPs) and promoter (16 SNPs plus two indels) regions of *CsGy5G023950*, none of these polymorphisms showed consistent association of alleles with fruit size/shape among 24 cucumber lines compared. *CsGy5G023950* was annotated to encode a P450 protein. Its Arabidopsis homolog, *AT2G26710*, encodes a member of the cytochrome p450 family (CYP72B1) that is involved in brassinosteroid (BR) biosynthesis/signaling and mediates responses to a variety of light signals including hypocotyl elongation and cotyledon expansion (e.g., [33,34]). In contrast, there was only one SNP (*A* in WI7167 vs. *G* in WI7200) inside the exon of *CsGy5G023910*, and the *A* allele was carried by WI7167, while the remaining 23 lines all had the alternate *G* allele. These preliminary data support *CsGy5G023910* as the most possible candidate for the fruit shape QTL *fs5.2* in cucumber although more evidence is needed to confirm the identity.

*CsGy5G023910* is a homolog of Arabidopsis *CRABS CLAW* (*CRC*), a member of the plant-specific YABBY transcription factor protein family. *YABBY* genes play many important roles in fruit development [35]. In Arabidopsis, the *crc-1* mutants cause the gynoecium to develop into a wider but shorter structure, and they further result in wider and shorter siliques, suggesting the involvement of *CRC* in silique/fruit size and shape control [35,36,37]. In melon, transgenic plants overexpressing the *CmCRC* gene results in elongated fruit [38]. Recently, Lian et al. [39] inferred that melon gene *MELO3C004493* encoding a YABBY transcription factor might be the candidate for a QTL controlling both fruit weight and length. These studies provide a possible link between the CRC function in regulating fruit size/shape in cucumber. How *CsCRC*/*FS5.2* regulates ovary/fruit growth and fruit size/shape is an interesting subject in future studies.

### 3.4. FS5.2-Dependent Fruit Size/Shape Variation May Involve Regulation of the Auxin/GA Biosynthesis/Signaling Pathways

Regardless of the nature of the candidate gene for *FS5.2*, like in many other fleshy fruits, the regulation of fruit size and shape by *FS5.2* is likely achieved through interaction and/or integration of phytohormone biosynthesis/signaling pathways to control the timing/duration of cell division or cell expansion [40,41,42]. Among major phytohormones the auxin and GA, as well as their interactions, play a more prominent important role in fleshy fruit set and growth. Cell division during fruit growth is particularly influenced by auxin signaling involving components such as ARF and Aux/IAA genes in the pathway (e.g., [43,44,45]). In addition, input from cytokinins (CKs), brassinosteroids (BRs), and ethylene (ET) can also influence cell division and expansion and fruit growth, which is well documented in cucumber. For example, the MADS box transcription factor CsFUL1 regulates fruit elongation via repressing *CsSUP* and downregulating *CsPIN1* and *CsPIN7* to regulate the cell division and expansion and the auxin accumulation in cucumber fruits, respectively [9]. The CsHEC1-CsOVATE regulatory module regulates cucumber fruit neck length variation via *CsYUC4*-mediated auxin biosynthesis [46]. On the other hand, SF1 ubiquitinates and degrades both itself and ACS2 to control ethylene synthesis for dose-dependent effect on cell division and fruit elongation in cucumber [6], whereas *SF2* encodes an HDC1 homolog and plays a role in regulating fruit cell proliferation through the HDAC complex to target the biosynthesis and metabolism of cytokinin and polyamines [7].

In this study, we examined the fruit growth and development dynamics from 6 days before anthesis until maturity (35 dpp) in both WI7237 and fs5.2-WI7237 NILs (Figure 2). Fruit growth in both lines exhibited a typical sigmoidal pattern; fruit development starts with a short fruit set phase, which is followed immediately by a rapid cell division phase until approximately 6 dpp, and then by an exponential expansion phase. This growth pattern is consistent with results from previous studies in cucumber (e.g., [3,30,47,48,49,50,51,52]). However, the fruit growth rate and duration of fruit elongation was significantly higher or longer in WI7237 than in fs5.2-WI7237 (Figure 2). Consistent with the longer but slimmer (narrower) fruit, microscopic examinations revealed longer cells and larger cell size (area) but fewer cellular layers of WI7237 fruits than fs5.2-WI7237 (Figure 3) suggesting that *FS5.2* regulates fruit shape by promoting longitudinal cell expansion but restricts lateral cell division. In both NILs, IAA content increased from −6 to −3 dpp and 0 to 6 dpp, but it was much higher in WI7237 than in fs5.2-WI7237 (Figure 4) suggesting that IAA plays a critical rule in initial cell division in both the ovary and the pollinated fruit. This is consistent with the roles of auxins in fruit set/growth of flesh fruits (reviewed in Fenn and Giovannoni [42]). Indeed, from the transcriptome data, several auxin-related genes were significantly differentially expressed between WI7237 and fs5.2-WI7237 including those for auxin biosynthesis (*YUCs*), homeostasis (*GH3.6*), signal transduction (*AUXs*), and polar transport (*PIN3* and *PILS7*) (Table S5). On the other hand, GA content showed a third spike in WI7237 from 9 to 12 dpp, indicating that GA may be more important in cell elongation during fruit growth. In the NIL transcriptomes, expressions of many genes involved in cell cycle, division, and cell wall were also significantly altered. These data strongly suggest that the auxin biosynthesis/signaling pathway plays a critical role in *FS5.2*-regulated cell division/expansion to control fruit size/shape development. At the same time, other phytohormones (GA in particular) also play a role in this process.

**Figure 7:**
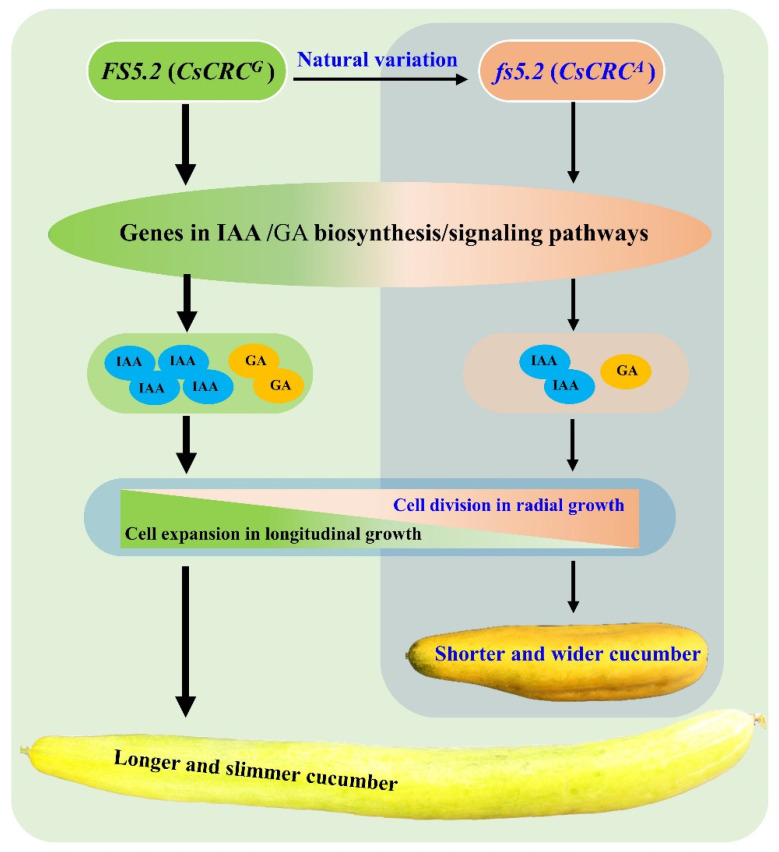
A working model depicting roles of *FS5.2* (*CsCRC*) in regulating cucumber fruit shape/size formation. A nonsynonymous SNP (*G* to *A*) in *CsCRC* might cause the changes in cucumber fruit shape/size from long and slim fruit in WI7237 (*FS5.2*, *CsCRC^G^*) to shorter and wider fruit in fs5.2-WI7237 (*fs5.2*, *CsCRC^A^*). *CsCRC^A^* mutation leads to lower contents of IAA and GA by regulating the expressions of genes involved in IAA and GA biosynthesis/signaling pathways, which promotes cell division (more cell layers) in radial fruit growth and inhibits cell expansion in longitudinal fruit growth, thus forming a shorter and wider fruit. Conversely, *CsCRC^G^* regulates the formation of longer and slimmer fruits in cucumber.

On the basis of the data presented herein, we propose a working model to explain the roles of *FS5.2*/*CsCRC* in regulating fruit size/shape formation in cucumber, which is illustrated in Figure 7. The *CsCRC^A^* mutation may lead to lower contents of IAA and GA by regulating the expressions of genes involved in IAA and GA biosynthesis/signaling pathways, which promotes cell division (more cell layers) in radial fruit growth and inhibits cell expansion in longitudinal fruit growth, thus forming a shorter and wider fruit. Conversely, *CsCRC^G^* increases the IAA and GA contents in cucumber fruit, yielding longer and slimmer fruit through promoting cell elongation longitudinally while decreasing the cell division horizontally.

## 4. Materials and Methods

### 4.1. Plant Materials

Two cucumber inbred lines WI7167 and WI7237, were used in the present study, which were the donor and the recipient (recurrent parent) in the development of near-isogenic lines (NILs) for the fruit size QTL *FS5.2*, respectively. WI7167, a selection from PI 618931, is a semi-wild XIS cucumber collected from Yunnan, China that sets nearly round fruits (length to diameter ratio or LD ≈ 1.0) [25]. WI7237 bears very long and slim fruit (>50 cm, LD ≈ 15.0) and is a landrace originally collected from China (Figure 1A and Figure 2A). The NIL pair, WI7237 and fs5.2-WI7237, was used for comparative morphological and histological observations, endogenous hormones measurement, transcriptome analysis, and fine genetic mapping in the present study.

All plant materials were grown in the Walnut Street Greenhouse (WSGH) or the field of the University of Wisconsin-Madison Hancock Agriculture Research Station (HARS), as well as the plastic tunnels in the Horticulture Farm of Northwest A&F University (HF-NWAFU) with standard cultivation managements.

Measurement of fruit size at immature and mature fruit stage of the two NILs was conducted in replicated trials in the greenhouses. For both WI7237 and fs5.2-WI7237, FL and FD were collected at multiple timepoints (i.e., −6, −3, 0, 3, 6, 9, 12, and 30 dpp) with three replications. At least three ovaries or fruits per replication were measured at each timepoint.

### 4.2. NIL Development for fs5.2

Marker-assisted backcrossing (MABC) was practiced, in which the *fs5.2* allele from WI7167 was introgressed into WI7237. Briefly, WI7167 was used as pollen donor to cross with the recurrent parent WI7237 (Figure 1). A single WI7237 × WI7167 F_1_ plant was backcrossed (BC) with WI7237. From BC_1_ to BC_4_, foreground selection was conducted using three markers including two flanking and one peak marker for *fs5.2*. At each BC generation, varying numbers of markers were also used for background selection for maximum recovery of the WI7237 genetic background. At BC_4_F_1_, plants that were heterozygous at the *fs5.2* region but homozygous for WI7237 alleles at all other marker loci were self-pollinated. The resulting BC_4_F_2_ plants carrying homozygous *fs5.2* allele from WI7167, fs5.2-WI7237, were near-isogenic with WI7237 at the target locus, which were used for all subsequent studies. Information on all markers used in the foreground and background selections is presented in Supplementary Table S1.

### 4.3. Fine Mapping of FS5.2

For fine mapping of the *fs5.2* major-effect QTL, segregating F_2_ populations from WI7237 × fs5.2-WI7237 were developed. We first examined segregation of fruit size in a small population with 86 F_2_ plants to confirm the effect of *fs5.2*. Then, recombinants were identified in a large F_2_ population with 864 individuals using two flanking markers 98C06 and 97C12. New polymorphic indel markers were explored in Illumina resequencing data (>15× coverage each) to genotype the recombinant plants. For marker discovery, Illumina sequencing reads were aligned to the Gy14v2.0 draft genome (http://cucurbitgenomics.org/organism/16, accessed on 9 August 2019), using the BWA (Burrows–Wheeler Alignment Tool) software package (https://bio-bwa.sourceforge.net/, accessed on 9 August 2019) [53]. Indel identification was performed by the SAM tools (https://samtools.sourceforge.net/, accessed on 9 August 2019) [53]. For Indels, only those with ≥3 bp differences between the two parental lines were utilized for primer design with Primer3web (http://bioinfo.ut.ee/primer3–0.4.0/, accessed on 10 October 2019).

DNA extraction, PCR amplification of molecular markers, and gel electrophoresis were conducted as described in [54]. Information on all markers used in genetic mapping is provided in Table S1.

### 4.4. Comparative Histological Observation of Ovary/Fruit Development in NILs

We conducted histological investigation of ovary/fruit development in WI7237 and fs5.2-WI7237 at 0, 12, and 30 days post pollination (dpp). The samples were fixed, embedded, sectioned with 8 μm in thickness, and dewaxed as described by Zhao et al. [9]. These samples were examined and imaged under an Olympus BX51 microscope. Cell length and width were measured in both cross-sections and longitudinal (vertical) sections of each sample using ImageJ (https://imagej.nih.gov/ij/, accessed on 8 September 2020). The measurements were made at three sites of each tissue for at least three sections from each fruit. The number of cells measured for each line at each stage varied from 77 to 187.

### 4.5. Transcriptome Analysis

We conducted RNA-Seq to explore the gene regulatory network associated with *fs5.2* on fruit size/shape in cucumber. Total RNA was extracted from the ovaries on anthesis day of the two NILs with the RNeasy Plant Mini Kit (Qiagen, Venlo, The Netherlands). There were two biological replications per sample. RNA-Seq was performed using Illumina Hi-Seq 2000. The paired-end (150 bp) clean reads were mapped to the Gy14v2.1 reference genome with TopHat (http://ccb.jhu.edu/software/tophat/index.shtml, accessed on 9 October 2021), and the transcripts were assembled with Cufflinks (http://cole-trapnell-lab.github.io/cufflinks/, accessed on 9 October 2021). Transcript abundance was calculated on the basis of the ratio of FPKM (fragments per kilobase of transcript per million mapped reads). Differentially expressed genes (DEGs) were identified through DESeq2 (http://bioconductor.org/packages/stats/bioc/DESeq2/, accessed on 9 October 2021) with the *p*-value <0.05 as the significance cutoff. GO enrichment and KEGG analysis of DEGs were performed at CuGenDBv2 (http://cucurbitgenomics.org/v2/, accessed on 9 October 2021) and by clusterProfiler [55].

### 4.6. Endogenous Hormones Measurement

We measured endogenous hormones in the ovary/fruit of the two NILs at −6, −3, 0, 3, 6, 9, and 12 days post pollination (dpp). Whole ovaries or young fruit at −6, −3, 0, and 3 dpp were directly used for hormone quantitation. For each biological sample, 3–5 fruits from different plants were pooled. Each sample at 6, 9, and 12 dpp was a mixture of the top (stem end), middle, and bottom (flower end) sections. There were three biological replicates for each sample. Collected samples were flash frozen in liquid nitrogen and kept at −80 °C freezer for hormone analysis.

The contents of auxin (indole-3-acetic acid, IAA), cytokinin (trans-zeatin riboside, tZR), and gibberellin (GA_3_) were measured using the procedures described in Pan et al. [56] and Farrow and Emery [57]. Briefly, the tissue (1–2 g) was ground into fine powder with a mortar and pestle in liquid nitrogen. One gram of sample was transferred into a 50 mL tube filled with 10 mL of pre-chilled (−20 °C) extraction solvent (isopropanol, H_2_O, and concentrated HCl = 2:1:0.002, *v*/*v*). The mixture was incubated at −20 °C for 16 h, and centrifuged at 4 °C, 10,000 rpm for 15 min. The supernatant was transferred to a new tube, and 1.5 volumes of dichloromethane (~30 mL) was added to the tube. The mixture was shaken for 1 h at 4 °C, and then centrifuged for 15 min at 10,000 rpm. The organic (hormone) phase was collected into a round-bottom flask and evaporated with a rotary evaporator under vacuum at 38 °C. The dry residue was dissolved in 1 mL of methanol and purified using a 0.22 μm syringe filter. The purified hormones were measured using HPLC–ESI-MS/MS (QTRAP 5500, AB Sciex).

### 4.7. Statistical Analysis of Data

All the collected phenotypic, histological, and hormonal data were firstly processed using Microsoft Excel 2016. The segregating ratio test of phenotypic or genotypic data was performed using the *χ*^2^ test in R (Version 4.0.3) with Rstudio (https://rstudio.com/, accessed on 10 August 2022). The Wilcoxon rank sum test (performed in R 4.0.3) was used to check the significant differences of histological data between two NILs. The statistical significance of fruit size (length and width) and hormonal data between WI7237 and fs5.2-WI7237 was determined using the Student’s *t* test (performed in Microsoft Excel 2016).

## Data Availability

All data pertinent to the reported work are provided in the manuscript or in the supplemental online materials. The complete raw reads data for RNA-Seq reported in this study were deposited to NCBI under BioProject accession PRJNA868393.

## Supplementary Materials

The supporting information can be downloaded at: https://www.mdpi.com/article/10.3390/ijms232113384/s1.
